# Development and validation of a genre-based second language (L2) writing self-efficacy scale

**DOI:** 10.3389/fpsyg.2023.1181196

**Published:** 2023-06-07

**Authors:** Jianhua Zhang, Lawrence Jun Zhang, Ye Zhu

**Affiliations:** ^1^School of Foreign Languages, Sichuan University of Arts and Science, Dazhou, China; ^2^Faculty of Education and Social Work, University of Auckland, Auckland, New Zealand; ^3^State Language Commission of China, Institute of Linguistics, Shanghai International Studies University and China Center for Language Planning and Policy Studies, Shanghai, China

**Keywords:** development of a genre-based L2 writing self-efficacy scale, genre characteristics, second language (L2), writing self-efficacy, psychometric quality, foreign language

## Abstract

Writing self-efficacy serves as one of the essential motivational factors in L1 and L2 writing, which has been measured by a series of scales in L1 and L2 contexts. However, the issue of task specificity was not resolved appropriately. This study aims to tackle this issue by entailing the genre characteristics of L2 writing tasks through developing a genre-based L2 writing self-efficacy scale with pertinent items. The new scale was designed with reference to the available research into writing self-efficacy. Its factorial structure was examined by structural equation modeling. Convergent validity and discriminant validity of the scale were examined by taking into consideration the average variance extracted and composite reliability for each individual factor involved in the scale, whereas the predictive validity of the scale was computed through regression analysis. Results show that the genre-based L2 writing self-efficacy scale demonstrated sound psychometric qualities. Theoretical and pedagogical implications of these research findings are discussed.

## 1. Introduction

Motivational factors play an essential role in the models of writing (e.g., Boscolo and Hidi, 2007; Hayes, 2012; Graham, 2018; Zhang, 2022). Writing self-efficacy has been widely acknowledged as one of the motivational factors in the first language (L1) and second or foreign language (henceforth referred to as L2) writing (Chen and Zhang, 2019; Chen et al., 2021) and is conceptualized as a multidimensional construct. For instance, Pajares and Valiante (1999) proposed that writing self-efficacy entailed ideation self-efficacy, writing convention self-efficacy, and self-regulation self-efficacy, whereas Teng et al. (2018) conceptualization included linguistic self-efficacy, self-regulation self-efficacy, and performance self-efficacy. Scholars have developed and validated scales or questionnaires for gaging writing self-efficacy in the L1 and L2 contexts due to the significance of self-efficacy in the learning process (e.g., Shell et al., 1989; Pajares and Valiante, 1999; Bruning et al., 2013; Teng et al., 2018; Sun and Wang, 2020). Bandura (2006) pointed out that items in self-efficacy scales or questionnaires are supposed to represent specific task demands. Notwithstanding, the available scales for measuring L1 and L2 writing self-efficacy have not dealt with the issue of task specificity appropriately. The employment of these scales might not lead us to gain a complete understanding of learners’ writing self-efficacy. Informed by the multidimensional perspective of writing self-efficacy, the current study is intended to tackle this issue by incorporating one of the vital writing task features (i.e., genre characteristics) in the development of items in the writing self-efficacy scale.

## 2. Literature review

### 2.1. Conceptualization and dimensions of writing self-efficacy

#### 2.1.1. Self-efficacy

Self-efficacy has been widely conceived as a vital construct in psychology and sociology, where it acted as a basic mechanism in psychosocial functioning. Scholars interpreted self-efficacy from different perspectives (e.g., Kirsch, 1985; Bandura, 1986). From the perspective of reinforcement theory, Kirsch (1985) viewed it as “expectancy for success at a task on which success is perceived to be dependent on ability” (p. 5). Bandura, however, recast it within the framework of social cognitive theory. He defined it as “people’s judgment of their capabilities to organize and execute courses of action required to attain designated types of performance” (Bandura, 1986, p. 391). Bandura commented on Kirsch’s conceptualization of self-efficacy and criticized Kirsch’s misinterpretation of self-efficacy by stating, “Kirsch further misrepresents self-efficacy theory when he alleges that the theory postulates low perceived self-efficacy as the cause of fear, irrespective of the domain of activity” (1986, p. 370). Thus, Bandura’s conceptualization of self-efficacy has gained increasing popularity and has been widely utilized in various studies (e.g., Schunk, 1991; Zimmerman, 2000; Matthews, 2010; Zumbrunn et al., 2020).

Self-efficacy, as Zimmerman (2000) pinpointed, served as an essential motive for learning, which can effectively predict students’ motivation and learning achievement. It acts on students by influencing their choices, effort, persistence and perseverance, thought patterns, and emotional reactions (Pajares, 2003). Students could develop their self-efficacy beliefs on the basis of four kinds of information: mastery experience, vicarious experience, social persuasion, and physiological and emotional states (Bandura, 1997). Mastery experience can be viewed as an interpretation of one’s previous performance, which served as a base on which to form their beliefs about their performance capabilities in subsequent tasks. Vicarious experience is interpreted as observing others’ performance or modeling. For instance, instructors can model planning skills to learners in the educational context. Social persuasion means evaluations of one’s capability that others give, which can be either positive or negative. Positive evaluations can enhance students’ self-efficacy beliefs, whereas negative ones weaken them. Physiological and emotional states such as pause, anxiety, stress, and arousal indirectly inform one’s self-efficacy beliefs.

Another strand of exploring self-efficacy was rooted in self-regulated learning theory. According to Bandura (1986), self-regulatory factors act as a central player in human functioning. Self-regulation was conceived as “a metacognitive process that requires students to explore their own thought processes so as to evaluate the results of their actions and plan alternative pathways to success” (Usher and Pajares, 2008, p. 443). Self-regulatory strategies could facilitate students to succeed in their development. It was found that students’ utilization of self-regulatory strategies was mainly determined by their beliefs about their capabilities to do so (Zimmerman and Cleary, 2006).

#### 2.1.2. Writing self-efficacy

Research has established that self-efficacy influences human motivation and action (e.g., Schunk, 1991; Zimmerman, 2000; Pajares, 2003; Schunk and Zimmerman, 2007; Caprara et al., 2008; Usher and Pajares, 2008). It is widely recognized as an essential player in acquiring writing competence and skills (e.g., McCarthy et al., 1985; Pajares and Johnson, 1994; Pajares and Valiante, 1997, 1999; Pajares et al., 1999). Writing self-efficacy refers to students’ perception of the writing capabilities that they possess to complete writing tasks (Pajares, 2003). It involves students’ judgement of “various composition, grammar, usage, and mechanical skills” (Pajares and Johnson, 1996, p.166). After summarizing numerous previous studies, Bruning et al. (2013) proposed a model of writing self-efficacy that included three focal dimensions: ideation, or idea generation; writing conventions, or translating ideas into words and sentences; and self-regulation, or the management, monitoring, and evaluation of writing processes. Ideation self-efficacy paid more attention to students’ confidence in their abilities to generate ideas. Idea generation served an essential role in the models of the writing process (e.g., Hayes and Flower, 1980; Hayes, 1996, 2012). It influenced other writing processes because of its cyclic nature. Writing convention self-efficacy is concerned with students’ confidence in following “a set of generally accepted standards for expressing ideas in writing in a given language” (Bruning et al., 2013, p. 28). For instance, the standards in English could cover spelling, punctuation, word, phrase, sentence, paragraph, discourse and their combinations for different populations. Self-regulation self-efficacy focused on students’ confidence in implementing self-regulatory skills successfully through the writing processes. It entails “a writer’s self-management and affective control but also involves judgments about cognitive and linguistic features as writing is being produced” (Bruning et al., 2013, p. 29). This model incorporated the thoughts accumulated about the writing process, writing and writing development. Thus, it was conceived as “consonant with writing process models emphasizing working memory’s centrality……as well as with other portrayals of writing and writing development……” (Bruning et al., 2013, p. 28). Additionally, Limpo et al. (2020) extended the conception of writing self-efficacy by adding two dimensions of handwriting and text genre, particularly for beginning writers in the L1 context.

Obviously, writing self-efficacy is a multidimensional construct. Given that there are huge differences between L1 and L2 writers, Teng et al. (2018) reconceptualized writing self-efficacy from three aspects of writing: linguistic, self-regulation, and performance. Their conceptualization was grounded in social cognitive theory and self-regulated learning theory and also informed by the difficulties that L2 writers encounter and the characteristics of L2 writing (Teng et al., 2018). Linguistic self-efficacy is concerned with students’ perception of their capability to retrieve words, translate ideas into sentences, and meet discourse requirements. Performance self-efficacy focuses on learners’ confidence in their abilities to complete a writing task in the instructional context. Self-regulation or self-regulatory efficacy was defined as “students’ perceived capability to execute metacognitive control in the learning-to-write process” (Teng et al., 2018, p. 23).

Although scholars held various conceptualizations of writing self-efficacy, they might have neglected some of the characteristics of writing tasks in their conceptualizations, thus not addressing the issue of task-specificity proposed by Bandura (2006). Furthermore, research has found that task characteristics (i.e., genre) could elicit learners’ distinctive writing performance (Yang et al., 2015, 2023; Yoon, 2021; Zhang and Cheng, 2021; Zhang and Zhang, 2021; Li and Zhang, 2022; Yang and Zhang, 2023). Therefore, it is suggested that the conceptualization of writing self-efficacy incorporate the aforementioned advances in L2 writing to demonstrate students’ perception of writing competence in different genres.

### 2.2. Measuring writing self-efficacy

Writing self-efficacy is defined as “students’ judgments of their competence in writing, specifically their judgments that they possess various composition, grammar, usage, and mechanical skills” (Pajares and Johnson, 1996, p.166). Three ways have been proposed to measure writing self-efficacy (Pajares, 2003). The first approach concentrates on the writer’s confidence in the specific skills they have mastered, such as grammar, usage, punctuation and storytelling. The second involves evaluating the confidence in accomplishing concrete writing tasks, for example, writing a term paper or a letter to a teacher or professor. The third approach focuses on assessing writers’ confidence in reaching specific performance criteria set by the course. The aforementioned ways of measuring writing self-efficacy reveal that it is a complex construct with different dimensions. Therefore, a multidimensional approach to writing self-efficacy would be necessary and vital for providing fine-grained information about writers’ self-perceived competence.

It is well-acknowledged that self-efficacy can be effectively measured by questionnaires/scales. Clear guidelines on the operationalization and measurement of self-efficacy beliefs were given by Bandura (2006), who emphasized: “self-efficacy assessment tailored to domains of functioning and task demands identify patterns of strengths and limitations of perceived capability” (p. 319). Therefore, developing self-efficacy scales entails fitting scale items with particular domains and specific demands of different tasks. In addition, Zimmerman (2000) stressed that: (1) self-efficacy measures entail performance capabilities rather than personal qualities; (2) self-efficacy measures should be administered to students prior to engaging in certain tasks. His emphasis might shed insights into the development of writing self-efficacy scales: the writing self-efficacy scale should incorporate items assessing learners’ confidence in performing specific writing tasks. Furthermore, it might also warn us of the timing of evaluating writing self-efficacy.

Research has revealed that writing self-efficacy exerted significant predictive effects on writing performance not only in L1 but also in L2 contexts (e.g., Pajares and Johnson, 1996; Prat-Sala and Redford, 2012; Teng et al., 2018; Sun and Wang, 2020). Several scales for writing self-efficacy have been designed and/or validated in L1 and L2 contexts. Pajares (2003) summarized three ways of measuring writing self-efficacy. The first way focused on evaluating students’ confidence in specific writing skills. They were operationalized as successful performance in using grammar, showing mastery of usage, writing a composition, and demonstrating mechanical writing skills (e.g., Shell et al., 1989; Pajares and Johnson, 1996), as specific story-writing skills (Graham and Harris, 1989), and as writing skills selected by teachers that were suitable for relevant tasks (e.g., Pajares and Valiante, 1997, 1999, 2001; Pajares et al., 1999). The second way entailed evaluating students’ confidence to complete specific writing tasks, for instance, a term paper and a letter (e.g., Shell et al., 1989; Pajares and Johnson, 1994). The third way involved the integration of the first and the second ways, thus assessing students’ confidence in demonstrating both specific writing skills and completing writing tasks.

Initially, Shell et al. (1989) created a scale for examining L1 students’ writing self-efficacy at a tertiary school. The scale was composed of two subscales: the task subscale measuring students’ confidence in performing writing tasks, and the skill subscale measuring their confidence in utilizing specific writing skills. They reported that scores of the skill subscale showed a sound predictive effect on writing achievement, but those of the task subscale did not. In other words, students’ writing skill self-efficacy can predict their writing performance, but their writing task self-efficacy can not. The scale was validated with young subjects of three different grades from primary and secondary school by Shell et al. (1995). They also found that writing skills self-efficacy rather than writing task self-efficacy exerted a significant predictive effect on writing achievement. The same result was reported in Pajares and Johnson (1994).

Later, the Writing Self-Efficacy Scale, developed by Pajares and Valiante (1999), has been widely utilized in studies relevant to writing self-efficacy because of its acceptable stability and internal consistency. Items in this scale were designed to measure middle school students’ perception of their confidence in how well they can utilize grammar, usage, composition, and mechanical writing skills in writing tasks. A 0–100 response format rather than a traditional Likert format was employed because the former showed better psychometric quality than the latter (Pajares et al., 2001). They found that writing skills self-efficacy served as a significant predictor of writing competence compared with variables such as writing self-concept, previous apprehension, and perceived value of writing. Subsequently, the underlying structure of this scale was examined through structural equation modeling by Pajares (2007), which uncovered that writing skills self-efficacy was composed of two factors: basic skills and composition skills.

While writing self-efficacy drew more and more attention from researchers, criticisms emerged regarding its evaluation. Bruning et al. (2013) criticized writing self-efficacy scales available at that time for the broad coverage of related writing skills and the lack of theoretical underpinnings. After reviewing a series of studies, Bruning et al. (2013) pointed out that “most writing self-efficacy measures, however, have broadly sampled writing-related skills and tasks, making them less than ideal for yielding information about writers’ self-efficacy for specific dimensions of writing” (p. 27). Besides, these instruments were found to be difficult to be related “directly to models of writing or to potentially writing-relevant psychological and language-related processes” (Bruning et al., 2013, p. 26).

As mentioned in the above section, Bruning et al. (2013) put forward a model of writing self-efficacy where self-efficacy beliefs were tied to writing models. The constructs in this model were examined and validated with middle and high school students through the Self-Efficacy for Writing Scale. This scale was composed of 16 items: ideation (5 items), conventions (5 items), and self-regulation (6 items). The proposed three-factor model of writing self-efficacy was confirmed, and the results suggested its generalizability.

All the aforementioned scales were designed to measure L1 students’ writing self-efficacy and adapted and modified to fit the specific research questions. To our knowledge, the Second Language Writer Self-Efficacy Scale was one of a few instruments available to evaluate students’ writing self-efficacy in a second/foreign language context. It was designed by Teng et al. (2018) on the basis of their conceptualization of writing self-efficacy. This scale consisted of 3 subscales with 20 items: linguistic self-efficacy (7 items), performance self-efficacy (7 items), and self-regulatory self-efficacy (6 items). Structural equation modeling was employed to validate the scale with Chinese-speaking EFL learners at the tertiary level. CFA revealed that the scale showed satisfactory psychometric qualities. Model comparisons demonstrated that the three-factor correlated model fits with the data collected better than the one-factor and three-factor uncorrelated models. However, items examining their conceptualizations were criticized for not having taken into consideration task specificity (Sun and Wang, 2020). Therefore, Teng et al. (2018) Second Language Writer Self-Efficacy Scale needs to be extended to incorporate features of writing tasks.

Recently, Sun and Wang (2020) developed a new scale named the Questionnaire of English Writing Self-Efficacy to assess writing self-efficacy in ESL or EFL contexts. Items in the questionnaire were drawn from the Self-Efficacy for Writing Scale and the Questionnaire of English Self-Efficacy, which were developed by Wang and Bai (2017) to evaluate EFL learners’ general English self-efficacy. The new scale examined five writing-related dimensions: ideation, organization, grammar and spelling, use of English writing, and self-regulation, each of which acted as a subscale. It was designed in a 7-point Likert response format. Cronbach’s alpha showed a good internal consistency of each subscale. CFA revealed that the five-factor model fitted the data. Notwithstanding, the Questionnaire of English Writing Self-Efficacy needs to be finetuned. This is because the issue of task specificity is left unsolved in that some items in the questionnaire are not directly relevant to the writing task included in their study, and features of a writing task, such as genre, are insufficiently covered. Moreover, the authors might not have given enough attention to the theories underpinning the design of the questionnaire.

As suggested by Bandura (2006), self-efficacy measurement should be tailored to cover task demands. In other words, the instruments of self-efficacy should be designed as task-specific. As known to us, writing tasks are defined in specific genres. Genres are characterized by different patterns of language use and rhetoric features (Wingate and Tribble, 2012). Genre features could impose constraints on written discourses, for example, employing certain linguistic patterns to achieve a persuasive purpose, thus constituting the high-level demands for writing essays, both in L1 and L2 contexts. Furthermore, it was found that learners demonstrated distinctive syntactic structures, phrases, and words across argumentative and narrative tasks (Ong and Zhang, 2010; Yang et al., 2015; Yoon, 2021). Thus, genre features of these tasks should be included as one of the essential components of writing self-efficacy instruments.

However, to our knowledge, few of the available instruments investigating writing self-efficacy took into consideration task characteristics, such as genre features. Employing these instruments to assess writing self-efficacy might result in, possibly, a partial understanding of learners’ self-belief in completing certain writing tasks; consequently, inappropriate teaching intervention can be rectified to achieve the expected teaching effectiveness and efficiency. Consequently, the validity and reliability of these instruments would be left questionable. To properly address this issue, therefore, it is imperative and reasonable to develop a new writing self-efficacy scale in relation to L2 writing contexts by incorporating task features of L2 writing, thus facilitating teachers, students and researchers to gain a more complete understanding of students’ writing self-efficacy and simultaneously providing a tentative solution to the issue raised by Bandura (2006) about the lack of task specificity in self-efficacy research.

## 3. Present study

This study aimed to develop a new scale for evaluating writing self-efficacy in the L2 context by incorporating task-specificity in relation to genre features of writing tasks. Therefore, we tried to answer three research questions:

1. What were the factors of the newly developed writing self-efficacy scale?
2. How did the factorial structure of the newly developed writing self-efficacy scale fit with the target subjects?
3. How did multi-dimensional writing self-efficacy predict L2 writing performance?

## 4. Methods

### 4.1. Participants

A total of 664 EFL students as convenient samples from a population of 50,000 at two medium universities in Western China were recruited to participate voluntarily by employing Slovin’s formula, namely, using the formula as shown here: *n* = N / (1 + Ne2); and the participants were divided into two groups: Sample A comprised 332 students, and Sample B consisting of 332 students. When participating in this study, the participants had studied English for at least 6 years since the majority of them started learning English while they were in junior high school, and their mother tongue is Mandarin Chinese. Their English writing instruction was embedded in the integrated English course. The participants ranged from the first year to the third year (55.42% freshmen, 15.663% sophomores, and 28.915% juniors), of which 59.04 per cent (*n* = 392) were females, and 40.96 per cent (*n* = 272) were males between the ages of 18 and 21 (Mean = 19.7). They were registered in the following majors: electronic engineering (*n* = 100, 15.06%), computer science (*n* = 114, 17.17%), education (*n* = 114, 17.17%), business (*n* = 176, 26.51%), administration (*n* = 96, 14.46%), and tourism (*n* = 64, 9.64%). The participants in both Sample A and Sample B were equivalent in grade, gender, and major/specialization distributions.

### 4.2. Measure development

Following the guide given by Bandura (2006) for constructing self-efficacy scales, we took the particular domain of functioning and task demands as the priority in this research. As implied by Pajares (2003), whether a self-efficacy scale is appropriate and adequate depends to a great extent on “the domain under investigation, its different features, the types of the capabilities it requires, and the range of the situations these capabilities might be applied” (p. 144). To establish the content validity of the scale appropriate for EFL writers at the tertiary level, we consulted and examined in a nuanced fashion some established instruments such as the Writing Skills Self-Efficacy Scale (WSES, Pajares and Valiante, 1999), the Writing Self-Regulatory Efficacy Scale (WSRES, Zimmerman and Bandura, 1994), the Self-Efficacy for Writing Scale (SEWS, Bruning et al., 2013) and the Second Language Writer Self-Efficacy Scale (SLWSES, Teng et al., 2018).

Initially, a total of 25 items relevant to EFL writing self-efficacy were produced. The content and face validity of these items were examined by three scholars who are well-published in local and international journals and based in the country where the study was conducted. Specifically, they scrutinized the theoretical rationale adopted here, evaluated whether the generated initial items matched the construct being targeted and measured, and checked whether the diction of the scale was clear and readable. The examination and evaluation of the items of this scale were performed in two rounds. The first round ended with the elimination of unnecessary items, the rewording of double-barreled items and the addition of items to remedy the obvious omissions.

Although a scale with a 0–100 response format has been proven to show stronger psychometric qualities in comparison with a traditional five−/seven-point Likert one in gaging self-efficacy beliefs (Pajares et al., 2001), the 0–100 response format was recently found to cause potential confusion for EFL learners when being employed to measure self-efficacy beliefs (Chen and Zhang, 2019; Chen et al., 2022). As a result, a compromise must be made, and then a 7-point Likert response format was adopted in this research. The finalized instrument containing 24 items was arranged in a logical fashion on the basis of the clusters of subcategories. A 7-point Likert scale with a gradation rating from 1 (*not at all true of me*) to 7 (*very true of me*) was adopted to explore the trait and state features of writing self-efficacy.

All the items of the current scale were developed in English and translated into Chinese when presented to the participants, guaranteeing that they can fully understand the items and avoid potential misunderstandings. The accuracy and equivalence of the translation were verified and backed up by means of translating and back-translating. Finally, the GL2WSS were then subjected to statistical tests to examine its construct validity.

### 4.3. English writing tests

Two writing tasks with a given topic were employed to examine the participants’ writing performance: a narrative writing task and an argumentative one. These tasks completely matched the genres covered in the newly developed GL2WSS and thus enabled the GL2WSS to be really task-specific. In addition, narrative writing was the first genre they learned to write, whereas argumentative writing was the one they practiced most because it was targeted in classroom assessments and national and international English tests.

Participants in this study were required to finish two English compositions of at least 150 words according to the given prompts (see Appendix A for details) within 40 min, respectively, in an online writing platform named Pigai in two rounds. The topic for the argumentative writing was chosen from the old item pool of the College English Test, Band 4 (CET 4), which showed high validity and reliability. In contrast, the topic for the narrative writing was designed as culturally inoffensive and closely related to participants’ daily life.

Jacobs et al.’s (1981) ESL Composition Profile, one of the established analytic scoring rubrics, was employed as the scoring criteria to assess participants’ writing performances. Jacobs et al.’s scoring rubric has been widely used in L2 writing studies to evaluate the writing proficiency levels of L2 students around the world by virtue of its relatively easy operationalization (e.g., Ong and Zhang, 2010; Huang and Zhang, 2020; Rahimi and Zhang, 2021). The rubric gages five aspects of written essays: content (i.e., including knowledge of the subject, development of a thesis, and relevance to topics), organization (i.e., including idea support, organization, and sequencing), language use (i.e., including constructions and grammatical errors), vocabulary (i.e., including word range, word choice and usage, form mastery, and word appropriacy) and mechanics (i.e., including mastery of conventions and error rate of spelling, punctuation, captalization and paragraphing), which are given different weights in the scoring scheme: content (30%), organization (20%), language use (25%), vocabulary (20%) and mechanics (5%). These aforementioned aspects can be rated at four rating levels: excellent to very good, good to average, fair to poor, and very poor.

### 4.4. Procedures

After signing the consent form, the participants were asked to complete the newly developed scale through an online survey platform named *Wenjuanxing* (literally translated as Questionnaire Star): The Genre-based Second Language Writing Self-Efficacy Scale (GL2WSS). As mentioned in the above section, all the items of the current questionnaire were developed in English and translated into Chinese when presented to EFL students to guarantee that the items could be fully understood and potential misunderstandings avoided.

The scale was given to students to elicit authentic context-based information. Before answering the questions in the questionnaire, all the students were told that their answers would not be judged as right or wrong on specific criteria and that they would be highly appreciated if they could provide accurate reports of writing self-efficacy. They were also told that their responses to the survey would not have any impact on their course grade at all. Before distributing the survey links to the participants, the researcher reviewed and clarified the instructions. Any doubts and comments from the participants were recorded and addressed during and after responding to the questionnaire. On average, respondents spent approximately 5–8 min finishing the questions in the questionnaire. The responses of students in Sample A were utilized to explore the factorial structure of the GL2WSS scale through exploratory factor analysis, while those from Sample B were to identify the relationship between the measured variables and the constructs in the GL2WSS scale.

After completing the online survey, students in Sample B were required to finish two writing tasks mentioned in the above section. A total of 664 essays were collected to investigate EFL learners’ writing achievement. Two EFL teachers who have taught English for at least 10 years in China and demonstrated excellent performance in scoring CET4 essays were invited to mark the collected essays independently under Jacobs et al. writing scoring rubrics. The inter-rater reliability between the raters was r_AB_ = 0.939, *p*_AB_ = 0.000 < 0.05 for argumentative writing, r_AB_ = 0.885, *p*_AB_ = 0.000 < 0.05 for narrative writing, indicating sound scoring reliability.

### 4.5. Data analysis

Data analysis included two phases: data preparation and instrument validation. Following the procedures proposed by Dörnyei and Taguchi (2010), data gathered from the questionnaire went through screening and cleaning first. In the data cleaning, the researcher performed corrections of as many errors and inaccuracies as possible, which included impossible answers, incorrectly entered answers, contradicting answers, and implausible data. The provided answers that indicated that participants who lacked effort, intentionally misbehaved, or responded in an inaccurate fashion were deleted from the database. The checking and cross-examination of missing data were done through a manual inspection first. Listwise deletion was adopted to remove all the cases of missing data using Microsoft Excel 2016. After data screening and cleaning, no participants were removed from participation in the pilot study. Therefore, 664 participants were retained for the final analysis in the pilot study.

The normality, linearity, and homogeneity of variance of the data were carefully checked prior to the actual multivariate analysis. As a common practice, Mardia’s kurtosis and/or skewness were adopted as a reference to check the multi-normality of the collected data. Generally speaking, when the critical ratio for Mardia’s skewness and kurtosis is less than 1.96, the data are assumed to be multi-normally distributed (Tavakoli, 2012); otherwise, the data would not show the property of multi-normality. The examination of Mardia’s kurtosis and skewness was conducted with the help of Stata 8.4.

In the instrument validation phase, the researcher adopted statistically rigorous procedures to scrutinize the reliability and the construct validity of the questionnaire in this study by running such analyses as factor analysis on the collected data. The reliability (also named internal consistency) of the questionnaire was measured by a reliability coefficient, Cronbach’s alpha. The construct validity was examined through two sub-constructs: convergent validity and discriminant validity.

Exploratory factor analysis (EFA) was utilized initially to explore the underlying factors or components of the newly designed scale, the Genre-based Second Language Writing Self-Efficacy Scale (GL2WSS). In EFA, the specific technique of a maximum-likelihood analysis with oblique rotation was employed (O’Connor, 2000). After that, confirmatory factor analysis (CFA) was applied to examine the relationship between the measured variables and the constructs or factors in the GL2WSS following the advice in Tavakoli (2012). IBM SPSS 25 was utilized to conduct EFAs on the aforementioned questionnaire, whereas MPlus 8.3, a latent variable modeling program offering various estimation methods for normal and non-normal data (Muthén and Muthén, 2018), was employed to perform CFAs on the questionnaire.

The convergent validity and discriminant validity of the questionnaire were examined by the combination of the average variance extracted and composite reliability for each individual factor involved in the questionnaire. The average variance extracted was employed to measure “convergence among a set of items representing a reflectively measured latent construct” (Hair et al., 2019, p.659), while composite reliability was to measure “reliability and internal consistency of the measured variables representing a latent construct” (Hair et al., 2019, p.659). The average variance extracted and the composite reliability were calculated with the help of an online tool, which is available at https://mlln.cn/. The critical value for the average variance extracted and the composite reliability are 0.5 and 0.7, respectively (Hair et al., 2019). In contrast, discriminant validity was examined by comparing the squared root of the average variance extracted and the correlational coefficient of the factors involved in the questionnaire. If the squared root of the average variance extracted is large than the correlational coefficients, it might indicate the sound discriminant validity of the questionnaire. Otherwise, it might indicate the opposite. In addition, the predictive validity of the GL2WSS scale was examined by performing regression analyses on factors in the scale and writing quality of argumentative and narrative writing, respectively.

## 5. Results

### 5.1. Descriptive statistics

In order to show the trend in the collected data, we need to see the distribution of participants’ responses to these items in the GL2WSS. As shown in Table 1, the mean scores of all 24 items involved in GL2WSS were in the range of 3.71 to 4.75, coupled with standard deviations ranging from 1.168 to 1.49. The values for the skewness and kurtosis for all the items ranged from −0.643 to 0.086 and from −0.581 to 0.331, respectively. According to the critical/cut-off values of +/− 3.0 and +/− 8.0 for skewness and kurtosis, respectively (Kline, 2016), the responses to the items showed the property of normal distribution.

**Table 1:** Descriptive statistics of GL2WSS.

	Mean	SD	Skewness	Kurtosis
Item 1	4.01	1.438	−0.029	−0.554
Item 2	4.01	1.441	−0.089	−0.581
Item 3	4.75	1.375	−0.643	−0.022
Item 4	4.28	1.359	−0.321	−0.241
Item 5	4.31	1.297	−0.361	−0.13
Item 6	4.49	1.3	−0.535	0.029
Item 7	4.44	1.221	−0.32	−0.061
Item 8	4.58	1.203	−0.345	−0.127
Item 9	4.38	1.207	−0.32	0.048
Item 10	4.42	1.233	−0.145	−0.345
Item 11	3.9	1.351	−0.049	−0.458
Item 12	3.71	1.49	0.086	−0.524
Item 13	3.77	1.422	−0.048	−0.401
Item 14	4.03	1.372	−0.053	−0.158
Item 15	4.2	1.28	−0.258	−0.108
Item 16	4.22	1.281	−0.244	−0.095
Item 17	4.55	1.189	−0.348	0.159
Item 18	4.43	1.233	−0.441	0.291
Item 19	4.39	1.225	−0.389	0.331
Item 20	4.37	1.231	−0.364	0.29
Item 21	4.4	1.27	−0.252	0.04
Item 22	4.25	1.205	−0.105	0.141
Item 23	4.17	1.246	−0.269	0.017
Item 24	4.17	1.168	−0.413	0.304

### 5.2. Factors extracted through exploratory factor analysis

The sampling adequacy was verified by the Kaiser-Meyer-Olkin (KMO) measure, the result of which (KMO = 0.944) shows that the valid sample size of 332 was sufficient for factor analysis. The strength of the correlations between items in the GL2WSS scale was measured by Bartlett’s test of sphericity, the result of which (*df* = 276, *p* < 0.001) indicates that these correlations were large enough to run factor analysis. Compared with other estimation methods, the maximum likelihood estimation is advantageous because it “allows for the computation of a wide range of indexes of the goodness of fit of the model, permits statistical significance testing of factor loadings and correlations among factors and the computation of confidence intervals” (Fabrigar et al., 1999, p. 277). Therefore, the maximum likelihood estimation was conducted on all 24 items via oblique rotation to extract factors. Following (O’Connor’s 2000) SPSS commands for parallel analysis, four predominant factors were extracted from the maximum likelihood estimation, explaining 63.442% of the cumulative variance. The four-factor solution was further examined to eliminate unsatisfactory items, including hyperplane items and some irrelevant items with low loading and complex loading. Following the recommended benchmark (+/− 0.5) for interpretability (Comrey and Lee, 2016), we retained the items with loading larger than 0.5. Eight items (items 1, 10, 15, 16, 17, 18, 20, 24, on the initial list) were eliminated because of complex loading (items that load at 0.5 or higher on two factors). Therefore, the other 16 items with loading larger than the benchmark were retained as the final version of the GL2WSS scale.

The revised GL2WSS scale, including 16 retained items, was re-assessed by employing the maximum likelihood estimation with oblique rotation, and the four-factor solution was confirmed (KMO = 0.929, *df* = 171, *p* < 0.001), explaining 64.852% of the total variance. No hyperplane items or items with complex and low loading were detected in the revised GL2WSS scale. Through the thematic analysis of items grouped around each factor, four categories of writing self-efficacy were identified and labeled: Factor 1 was labeled as *Linguistic Self-Efficacy* (47.318% variance); Factor 2 as *Classroom Performance Self-Efficacy* (7.617% variance); Factor 3 as *Genre-Based Performance Self-Efficacy* (5.663% variance); and Factor 4 as *Self-Regulatory Self-Efficacy* (4.254% variance). The final version of the 16-item GL2WSS scale and standardized factor loadings for those items, together with Cronbach’s alpha coefficients, are shown in Table 2.

**Table 2:** Factor loadings for three-factor model after EFA and internal reliability.

	Items	Factor loading				α
		1	2	3	4	
Linguistic self-efficacy (LS)	Item 3	0.765				0.884
	Item 4	0.757				
	Item 5	0.754				
	Item 6	0.654				
	Item 2	0.514				
Class performance self-efficacy (CPS)	Item 12		−0.942			0.901
	Item 13		−0.902			
	Item 14		−0.643			
	Item 11		−0.578			
Genre-based performance self-efficacy (GPS)	Item 21			0.844		0.903
	Item 23			0.822		
	Item 22			0.81		
	Item 19			0.591		
Self-regulatory self-efficacy (SRS)	Item 9				−0.84	0.858
	Item 8				−0.74	
	Item 7				−0.53	

α = Cronbach’s alpha coefficient.

Table 2 shows that Cronbach’s alpha coefficients for the four factors were 0.884 for linguistic self-efficacy, 0.901 for classroom performance self-efficacy, 0.903 for genre-based performance self-efficacy, and 0.858 for self-regulatory self-efficacy, which were larger than the critical value of no less than 0.70 for satisfactory reliability.

### 5.3. Four-factor correlated models through confirmatory factor analysis

Before conducting CFA on all 16 items in the GL2WSS scale, we examined the multivariate normality by using Mardia’s kurtosis value, whose critical ratio is 1.96 (Raykov and Marcoulides, 2008). The critical ratio of Mardia’s kurtosis value in the current study was 56.517, which is larger than the cut-off point, suggesting that the responses to the GL2WSS scale are multivariate non-normal. Therefore, the maximum likelihood estimation with robust standard errors proposed in Mplus (Muthén and Muthén, 2018) was employed here to examine the factorial structure of the GL2WSS.

The Four-Factor Models were constructed on the basis of the result of EFA in the above section. It specified 16 items into four distinct but correlated writing self-efficacy. Initially, Model 1 was generated where each indicator was constrained to load only the factor it was designed to measure; covariance for each factor pair was freely estimated, and measurement error for each indicator was also freely estimated and uncorrelated. Model 2 was then constructed by correlating the factors in the scale. Models 1 and 2 were subjected to omnibus fit statistical analyses, and fit indices for them were compared, as shown in Table 3.

**Table 3:** Fit indices for models 1 and 2.

	*x^2^*	*df*	CFI	TLI	RMSEA	AIC	BIC
Model 1	327.837	146	0.930	0.918	0.061	17242.666	17482.389
Model 2	210.608	131	0.969	0.960	0.043	17083.864	1738.664

**Figure 1:**
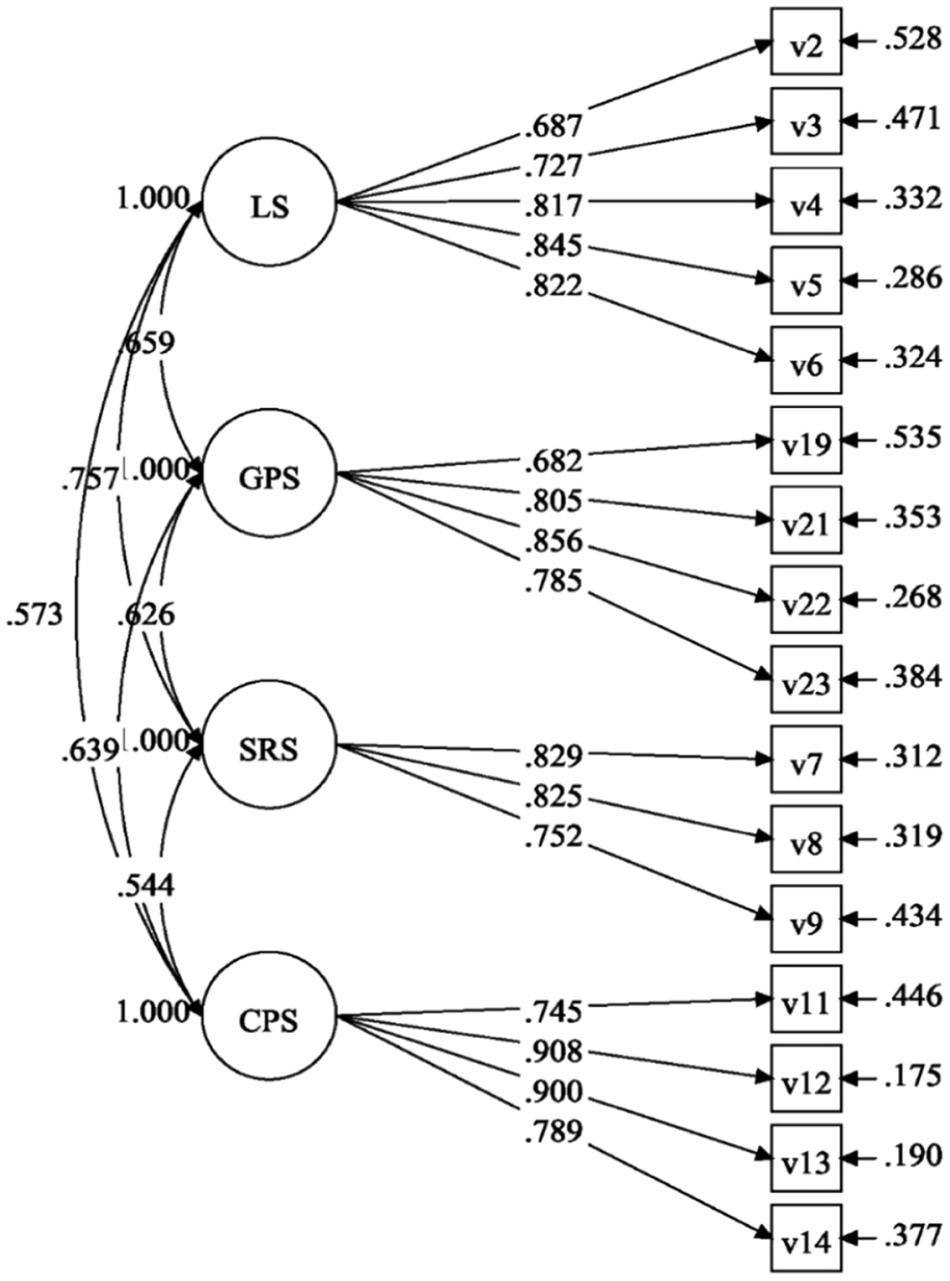
The four-factor correlated model of writing self-efficacy scale. GPS, genre-based performance self-efficacy; LS, linguistic self-efficacy; SRS, self-regulatory self-efficacy; CPS, classroom performance self-efficacy.

Fit indices in Table 3 show that Model 2 demonstrated a more satisfactory model fit than Model 1 (*𝑥*^2^ = 210.608; *df* = 131; *p* < 0.001; *𝑥*^2^ /𝑑*f* = 1.608; CFI = 0.969; TLI = 0.96; RMSEA =0.043 [0.032, 0.053]). Model 2, the final Four-factor Correlated Model, is presented in Figure 1.

Table 4 shows that the parameter estimates for all 16 items were statistically significant at *p* < 0.001, and standardized loadings of the items on the corresponding latent factors ranged from 0.687 to 0.908, which are higher than the recommended value of 0.50, indicating the large effect size (Raykov and Marcoulides, 2008). This might suggest that the latent factors showed sound representativeness of the corresponding items. The average variance extracted values for genre-based performance self-efficacy, linguistic self-efficacy, self-regulatory self-efficacy, and classroom performance self-efficacy were larger than 0.5, while their composite reliability estimates were higher than 0.8, both of which might indicate the sound convergent validity of the GL2WSS scale.

**Table 4:** CFA standardized regression weights for the four-factor correlated model of writing self-efficacy.

	Estimate	S.E.	*p*	AVE	CR
v2 ← LS	0.687	0.041	***	0.6086	0.8854
v3 ← LS	0.727	0.036	***		
v4 ← LS	0.817	0.034	***		
v5 ← LS	0.845	0.024	***		
v6 ← LS	0.822	0.027	***		
v7 ← SRS	0.829	0.03	***	0.5889	0.8505
v8 ←← SRS	0.825	0.035	***		
v9 ← SRS	0.752	0.04	***		
v11 ← CPS	0.745	0.037	***	0.6671	0.889
v12 ← CPS	0.908	0.018	***		
v13 ← CPS	0.9	0.021	***		
v14 ← CPS	0.789	0.036	***		
v19 ← GPS	0.715	0.046	***	0.5639	0.8857
v21 ← GPS	0.785	0.041	***		
v22 ← GPS	0.766	0.044	***		
v23 ← GPS	0.727	0.045	***		

*** = *p* < 0.001; S.E, standard error; AVE, average variance extracted; CR, composite reliability; GPS, genre-based performance self-efficacy; LS, linguistic self-efficacy; SRS, self-regulatory self-efficacy; CPS, classroom performance self-efficacy.

Table 5 demonstrates that latent factors of genre-based performance self-efficacy, linguistic self-efficacy, self-regulatory self-efficacy, and classroom performance self-efficacy had a significant correlation at *p* < 0.01. Although the correlations across the above latent factors were more than 0.5, they were less than square roots of average variance extracted values for latent factors. All these estimates might suggest that latent factors had correlations to each other and a degree of differentiation, indicating a good discriminate validity of the GL2WSS scale.

**Table 5:** Square root of AVEs and correlation matrix of four factors.

	LS	GPS	SRS	CPS
LS	0.6086			
GPS	0.626	0.5639		
SRS	0.752	0.597	0.5889	
CPS	0.63	0.657	0.62	0.6671
Square root of AVE	0.78	0.751	0.767	0.817

Average variance extracted values were shown in diagonals. GPS, genre-based performance self-efficacy; LS, linguistic self-efficacy; SRS, self-regulatory self-efficacy; CPS, classroom performance self-efficacy.

### 5.4. Predictive value of GL2WSS

Stepwise regression analysis was employed to examine the predictive validity of the GL2WSS scale. We listed the scores of argumentative and narrative writing tasks, respectively, as dependent variables. Specifically, stepwise regression analyses were carried out where only genre-based performance self-efficacy entered into the regression model and other three factors (i.e., linguistic self-efficacy, self-regulatory self-efficacy, and classroom performance self-efficacy) did not enter into the model because of the low predictive values. Besides, another regression analysis was conducted to examine the predictive effects of overall writing self-efficacy on the quality of both argumentative and narrative essays. The results of these analyses are shown in Table 6.

**Table 6:** Predictive effects of GL2WSS on writing quality.

Regression models and predictors	Writing quality							
	Argumentation				Narration			
	*B*	*β*	*t*	*p*	*B*	*β*	*t*	*p*
Regression	*R*^2^ = 0.053 *F* = 18.539 *p* = 0.000				*R*^2^ = 0.021 *F* = 6.917 *p* = 0.009			
Genre-based performance self-efficacy	0.359	0.231	4.306	0.000	0.121	0.143	2.630	0.009
Linguistic self-efficacy		0.038	0.578	0.564		−0.009	−0.138	0.890
Self-regulatory self-efficacy		−0.100	−1.56	0.120		−0.053	−0.783	0.434
Classroom performance self-efficacy		−0.014	−0.200	0.841		0.030	0.409	0.683
Regression	*R*^2^ = 0.035 *F* = 11.865 *p* = 0.001				*R*^2^ = 0.011 *F* = 4.838 *p* = 0.029			
Overall writing self-efficacy	0.087	0.186	3.445	0.001	0.037	0.12	2.20	0.029

It can be seen from the statistics in Table 6 that genre-based performance self-efficacy contributed to the writing quality of argumentative and narrative essays (*R*^2^_argumentation_ = 0.053, *p*_argumentation_ = 0.000; *R*^2^_narration_ = 0.021, *p*_narration_ = 0.009). Specifically, genre-based performance self-efficacy could explain 5.3% of the variability of the writing quality of argumentative essays but only 2.1% of that of narrative essays. In other words, students with higher levels of genre-based performance self-efficacy might write better argumentative and narrative essays than those with lower levels of genre-based performance self-efficacy. In contrast, other three factors generated insignificant predictive effects on the quality of the argumentative writing essays (*β*_linguistic self-efficacy_ = 0.038, *p = 0*.564; *β*_self-regulatory self-efficacy_ = −0.1, *p = 0*.12; *β*_classroom performance self-efficacy_ = −0.014, *p = 0*.841) and that of the narrative ones (*β*_linguistic self-efficacy_ = −0.009, *p = 0*.89; *β*_self-regulatory self-efficacy_ = −0.053, *p = 0*.434; *β*_classroom performance self-efficacy_ = 0.030, *p = 0*.683).

Additionally, Table 6 reveals that genre-based performance self-efficacy contributed to the writing quality of argumentative and narrative essays (*R*^2^_argumentation_ = 0.035, *p*_argumentation_ = 0.000; *R*^2^_narration_ = 0.011, *p*_narration_ = 0.029). It can be found from Table 6 that overall writing self-efficacy had significant predictive effects on the quality of the argumentative essays (*β* = 0.186, *p = 0*.001) and that of the narrative ones (*β* = 0.12, *p = 0*.029).

## 6. Discussion

This study aims to tackle the issue of task-specificity by incorporating genre characteristics into the design and development of items of the GL2WSS. The results of EFA and CFA in the above section support the factorial structure of the newly developed GL2WSS, including four categories of self-efficacy: genre-based performance self-efficacy, linguistic self-efficacy, self-regulatory self-efficacy, and classroom performance self-efficacy. Scores of four sub-constructs were calculated separately to reveal the level of participants’ perceived writing self-efficacy and collectively summed to show each student’s overall level of each sub-construct in terms of linguistics, self-regulation, task, and situation. The results might offer preliminary evidence for including task characteristics (e.g., genre features) in the conceptualization of writing self-efficacy. Therefore, it might provide a tentative solution to the issue of task-specificity proposed by Bandura (2006), thus enabling the writing self-efficacy scale to be task-specific.

The findings of this study might also provide initial evidence for extending the conceptualization of writing self-efficacy in the L2 context. The models specified by SEM confirmed the conceptualization of writing self-efficacy as four distinctive but correlated sub-constructs subsumed under the construct of writing self-efficacy: linguistic self-efficacy, self-regulatory self-efficacy, classroom performance self-efficacy, and genre-based performance self-efficacy. The first three sub-constructs were consistent with Teng et al. (2018) conceptualization of writing self-efficacy in the L2 context. Furthermore, as mentioned before, genre features could impose constraints on written discourses, for example, employing certain linguistic patterns to achieve the persuasive purpose, thus constituting the high-level demands for writing essays, both in L1 and L2 contexts. Therefore, incorporating genre features into the conceptualization of writing self-efficacy might cover the judgment of students’ confidence in meeting higher writing requirements that were left untouched in the previous studies concerning the development of writing self-efficacy scales. The finding that the GL2WSS scale demonstrated sound convergent validity might suggest that linguistic self-efficacy, self-regulatory self-efficacy, classroom performance self-efficacy, and genre-based performance self-efficacy were correlated. Moreover, the findings of this study also revealed that the GL2WSS scale demonstrated sound discriminate validity, which might suggest that four categories of self-efficacy entailed in the GL2WSS scale were conceptually and empirically distinguished. Accordingly, it can be concluded that the inclusion of genre characteristics into the conceptualization of writing self-efficacy is empirically validated. Meanwhile, the findings of this study also corroborated the multidimensional nature of writing self-efficacy in the L2 context. Overall, compared with previous studies (e.g., Teng et al., 2018; Sun and Wang, 2020), the findings of this study might advance our understanding of the multidimensional nature of writing self-efficacy by entailing task characteristics (i.e., genre features). Thus, writing self-efficacy could be conceptualized in terms of linguistic skills, self-regulation, tasks, and situations.

Furthermore, the results of this study indicated that compared with the other sub-constructs subsumed in the GL2WSS scale, genre-based performance self-efficacy had a significant predictive effect on the quality of argumentative and narrative essays in the L2 context. That is, students who are efficacious in genre-based performance might show better performance in writing argumentative and narrative essays than those who are not. The better predictive effect of genre-based performance self-efficacy could be attributed to the fact that the items tapping into this category of writing self-efficacy were more closely relevant or matched to the specific genre features of writing tasks. The genre features of argumentative and narrative essays were entailed in the development of the GL2WSS scale, while students were required to write essays in two genres: argumentation and narration. The match between the genre features of writing tasks and those entailed in the GL2WSS scale could facilitate students to make a more accurate judgement about their performance in certain writing tasks. Therefore, the findings of this study might provide another independent evidence for the predictive effects of writing self-efficacy on writing performance as reported in the literature. On the whole, the findings provided substantial evidence for the utility of the GL2WSS scale as an effective measurement of writing self-efficacy.

Additionally, the differences in the predictive effects of genre-based performance self-efficacy and overall writing self-efficacy on the quality of the argumentative essays and that of the narrative ones might be attributed to the practice effect. As mentioned before, argumentative writing has been set in national tests (e.g., College English tests and Tests for English majors in China) and international ones (e.g., The International English Language Testing System and The Test of English as a Foreign Language). Therefore, instructors would focus on the teaching of argumentative writing due to the washback effects of these tests. Naturally, argumentative writing tasks might be frequently assigned to students and they may practice them accordingly, thus their skills in writing argumentative essays being sharpened.

## 7. Conclusion

This study is designed to develop and validate a new scale for assessing writing self-efficacy in L2 contexts by incorporating genre features of writing tasks. Statistical analyses demonstrated that the newly developed GL2WSS scale demonstrated sound psychometric qualities, including good reliability, sound factorial structure, convergent validity, and discriminate validity. The findings of this study that the GL2WSS scale entailing linguistic self-efficacy, self-regulatory self-efficacy, classroom performance self-efficacy, and genre-based performance self-efficacy offered further evidence to support the multidimensional conceptualization of writing self-efficacy in a specific L2 context. The results of this study might also suggest that writing self-efficacy could be conceptualized in terms of linguistic skills, self-regulation, tasks, and situations, thus providing a tentative solution to the issue of task specificity of writing self-efficacy and offering initial evidence to support that writing self-efficacy is context sensitive.

The GL2WSS scale could be employed as a pedagogical tool in the classroom to facilitate teachers and students of EFL writing in assessing different aspects of writing self-efficacy. The inclusion of task-specific features such as text genre in the newly developed writing self-efficacy scale might help students make more accurate judgments of writing self-efficacy. The GL2WSS scale might offer students an opportunity to understand their writing capabilities from linguistic, classroom performance, genre-based performance, and self-regulatory aspects, all of which could motivate them to enhance their writing proficiency. Besides, teachers are advised to use this scale to know the profile of their students’ writing self-efficacy, which could facilitate teachers to adjust their writing instructions to engage students with achievement and enjoyment. The GL2WSS scale might be utilized (a) to elicit students’ writing self-efficacy to provide the guidelines for designing curriculum and teaching activities in writing courses; (b) to examine the underlying factors of writing self-efficacy; (c) to gage the effectiveness of teaching inventions; (d) to evaluate the preciseness of students’ judgments of writing competence and help them to align them if necessary.

Despite the fact that optimistic findings were generated from this study, several limitations should be recognized due to the constraints of experimental conditions, experimental methods and available resources. To begin with, the target population in this study were a sample of Chinese EFL writers from two medium-ranking universities, and the four-factor structure of writing self-efficacy may not be valid for other L2 cohorts. Therefore, the GL2WSS scale might need further validation and refinements to suit different populations in different learning contexts. Besides, we entailed the genre features of argumentative and narrative writing without involving other genres. Therefore, it is recommended that the characteristics of other genres be incorporated, or the genre features of the GL2WSS scale be tailored to match the writing tasks employed to examine students’ writing performance. In addition, there are three approaches available to examine criterion validity: retrospective validity, predictive validity, and concurrent validity (Horstmann et al., 2020). This study only focused on the predictive validity of the GL2WSS scale, thus leaving its retrospective validity and concurrent validity unexamined. Therefore, it might be interesting to investigate the extent to which writing self-efficacy and other motivational constructs, such as motive to write, might be conceptually and empirically distinguished or the relationship between writing self-efficacy and the criteria set previously.

## Data availability statement

The original contributions presented in the study are included in the article/supplementary material, further inquiries can be directed to the corresponding author.

## Ethics statement

The studies involving human participants were reviewed and approved by The University of Auckland Human Ethics Committee. The patients/participants provided their written informed consent to participate in this study.

## Author contributions

JZ and LZ conceived and designed the study. JZ collected and analyzed the data and drafted the manuscript, and all the authors revised and approved the manuscript. YZ and LZ finalized it. All authors contributed to the article and approved the submitted version.

## Funding

This work is funded by the Philosophy and Social Science Foundation of Shanghai, China (Grant no 2019BYY009).

## Conflict of interest

The authors declare that the research was conducted in the absence of any commercial or financial relationships that could be construed as a potential conflict of interest.

## Publisher’s note

All claims expressed in this article are solely those of the authors and do not necessarily represent those of their affiliated organizations, or those of the publisher, the editors and the reviewers. Any product that may be evaluated in this article, or claim that may be made by its manufacturer, is not guaranteed or endorsed by the publisher.

## Appendix A: writing prompts

1. Computers and the Internet have improved the efficiency and quality of learning for university students in China. Do you agree or disagree with the statement? Support your position with reasons. You should write at least 150 words.
2. Describe one of your unforgettable English learning experiences. You should write at least 150 words.

## Appendix B: genre-based L2 writing self-efficacy scale

In this part, we would like you to help us by answering the following questions concerning your writing self-efficacy. Please give your answers sincerely, as only this will guarantee the success of the investigation. Thank you very much for your help.

In the following section, we would like you to tell us how much you agree or disagree with the following statements by simply ticking (√) a number from 1 to 7. We are interested in your real situation and attitudes. Please do not leave out any of the items.

**Table:**

Not at all true of me	Not true of me	Slightly not true of me	Neutral	Slightly true of me	True of me	Very true of me
1	2	3	4	5	6	7

For example:

I like English movies. 1 2 3 4 5 6 7.

**Table:**

2. I can correctly use all parts of speech (e.g., nouns, verbs, adjectives, etc.) in writing.	1 2 3 4 5 6 7
3. I can write a simple English sentence with grammatical structure.	1 2 3 4 5 6 7
4. I can write compound and complex English sentences with grammatical structure.	1 2 3 4 5 6 7
5. I can write a good English paragraph with topic sentence or main idea.	1 2 3 4 5 6 7
6. I can write an English composition with a clear organisation or structure.	1 2 3 4 5 6 7
7. I can realise my goal to improve my English writing.	1 2 3 4 5 6 7
8. I can think of my goals before English writing.	1 2 3 4 5 6 7
9. I can think of different ways to help me to plan before English writing.	1 2 3 4 5 6 7
11. I can get an excellent grade for writing in the English course.	1 2 3 4 5 6 7
12. I can understand the most difficult writing material presented in the English course.	1 2 3 4 5 6 7
13. I can understand the basic concepts of writing taught in the English course.	1 2 3 4 5 6 7
14. I can understand the most complex material of writing presented by the instructor of the English course.	1 2 3 4 5 6 7
19. I can write an English narrative that includes several things that happened to the characters.	1 2 3 4 5 6 7
21. I can write an English narrative that describes clearly how the events develop.	1 2 3 4 5 6 7
22. I can write an English argumentation that includes arguments.	
23. I can write an English argumentation that includes sufficient evidences to support the arguments.	1 2 3 4 5 6 7
